# Popliteal pseudoaneurysm after FOLFOX chemotherapy for metastatic colorectal cancer

**DOI:** 10.1016/j.ijscr.2019.08.028

**Published:** 2019-08-31

**Authors:** Luka Cosic, Mayo Theivendren, Manfred Spanger, Laurence Weinberg

**Affiliations:** a Department of Anaesthesia, Austin Hospital, Heidelberg, Victoria, 3084, Australia; b Department of Vascular Surgery, Austin Hospital, Victoria, 3084, Australia; c Department of Radiology, Box Hill Hospital, Box Hill, Victoria, 3128, Australia; d Department of Surgery, Austin Health, University of Melbourne, Victoria, 3084, Australia

**Keywords:** Popliteal artery, Aneurysm, Endovascular, Chemotherapy, Colorectal, Cancer

## Abstract

**Introduction:**

Popliteal artery aneurysms are a rare occurrence in the general population. We present the case of a male who developed a popliteal artery pseudoaneurysm following chemotherapy for metastatic colorectal cancer.

**Case presentation:**

A 49-year old male presented with a popliteal artery pseudoaneurysm after completing four two-weekly cycles of FOLFOX chemotherapy. There was no history of infection, knee trauma, inflammatory diseases, or any family history of cardiovascular disease or aneurysms. Examination revealed a tender pulsatile mass in the right popliteal fossa with calf oedema. Computed tomography angiography demonstrated a right popliteal pseudoaneurysm, that was treated with endovascular stent grafting.

**Discussion:**

Anecdotal evidence suggests a link between chemotherapy and the rapid development of abdominal aortic aneurysms exists. Aneurysms have been reported following cisplatin and 5-fluorouracil treatment and trans-arterial administration of irinotecan, a key component of chemotherapy. Chemotherapeutic agents have also been shown to compromise the integrity of the vascular wall through apoptosis of endothelial and smooth muscle cells. In our case, the pseudoaneurysm developed acutely after treatment with FOLFOX, therefore a mechanistic association is plausible.

**Conclusion:**

Differentiating aneurysms as false (pseudo) or true is important to help determine the underlying aetiology. Common causes of pseudoaneurysms include arterial blunt or penetrating trauma. True aneurysms commonly develop from inflammatory atherosclerosis, however mycotic infection, inflammatory arteritis, and entrapment syndrome should be excluded. There may be some evidence to suggest a genetic predisposition to popliteal artery aneurysms. Anecdotal evidence suggests a weak association between chemotherapy and aneurysm progression, warranting further investigation into a causative link.

## Highlights

• Popliteal artery aneurysms are a rare occurrence and can result in limb-threatening ischaemia.
• Popliteal artery aneurysms can be differentiated into false or pseudonaneurysms and true aneurysm based on aetiology.
• Anecdotal evidence suggests a weak association between chemotherapy and aneurysm development.

## 1 Introduction

Popliteal artery aneurysms (PAAs) represent a serious disease process with potentially devastating complications and sequelae. Both true and false PAAs have historically been associated with high rates of limb loss, although with prompt management in the era of modern medicine these complications are rarely seen. However, limb-threatening ischaemia following thrombus formation and distal embolization remains a concern surrounding the development of PAAs [1,2]. Appropriate treatment of PAAs requires prompt definitive management with either surgical resection or endovascular stenting [3].

Although rare, with a reported incidence of approximately 1 in 100,000 for true aneurysms, and fewer still for pseudo or false aneurysms [4,5], PAAs are easily recognised clinically as tender pulsatile masses in the popliteal fossa, associated with popliteal fossa discomfort or pain. Aetiology is markedly different for true and false aneurysms. Pseudoaneurysm (also known as a false aneurysm) is a collection of blood that forms between the two outer layers of an artery, the tunica media and the tunica adventitia. Typical aetiology for pseudoaneurysm include a history of arterial trauma such as arterial access for catheterization, and blunt or penetrating trauma [6]. In contrast, true aneurysms are bound by all three layers of the vessel wall (tunica intima, media and adventitia) and typically develop from inflammatory atherosclerosis [7,8]. Other less common causes of true aneurysms include mycotic infection, inflammatory arteritis, and entrapment syndrome, or as the result of mechanical stress from repeated movement of the knee [[9], [10], [11]]. There is some evidence to suggest a genetic predisposition to PAAs [12,13] and additionally, anecdotal evidence exists suggesting a relationship between chemotherapy and aneurysm progression [[14], [15], [16]].

We present the case of a male who developed a popliteal artery pseudoaneurysm immediately following a course of chemotherapy for metastatic colorectal cancer. We explore the plausible association between the patient’s chemotherapy regime and development of popliteal artery pseudoaneurysm. This report was prepared according to the SCARE statement for reporting surgical case reports [17].

## 2 Presentation of case

A 49-year old Caucasian male, weight 72 kg, height 178 cm, presented with sudden onset right popliteal fossa discomfort associated with oedema of the right leg. He had no symptoms of claudication or critical limb ischaemia. His symptoms presented 5 days after completing four two-weekly cycles of FOLFOX6 (modified) adjuvant chemotherapy for stage IV colorectal cancer with liver metastases. Prior to FOLFOX chemotherapy, the patient failed to respond to 2 months of FOLFIRI chemotherapy, which included irinotecan (180 mg/m^2^ IV over 90 min) concurrently with folinic acid (400 mg/m^2^ IV over 120 min, followed by fluorouracil (400 mg/m^2^ IV bolus) then fluorouracil (2500 mg/m^2^ intravenous infusion over 46 h). This cycle was repeated every two weeks for 4 months. Each cycle of FOLFOX chemotherapy consisted of a day of treatment separated by 14 rest days. Treatment on Day 1 included calcium folinate (Leucovorin®) 50 mg IV bolus, oxaliplatin 85 mg/m^2^ IV infusion, fluorouracil 400 mg/m^2^ IV, and fluorouracil 2400 mg/m^2^ continuous IV via pump over 46 h. The patient had no history of mycotic infection, knee trauma, or inflammatory diseases. There was no family history of cardiovascular disease or aneurysms.

**Fig. 1:**
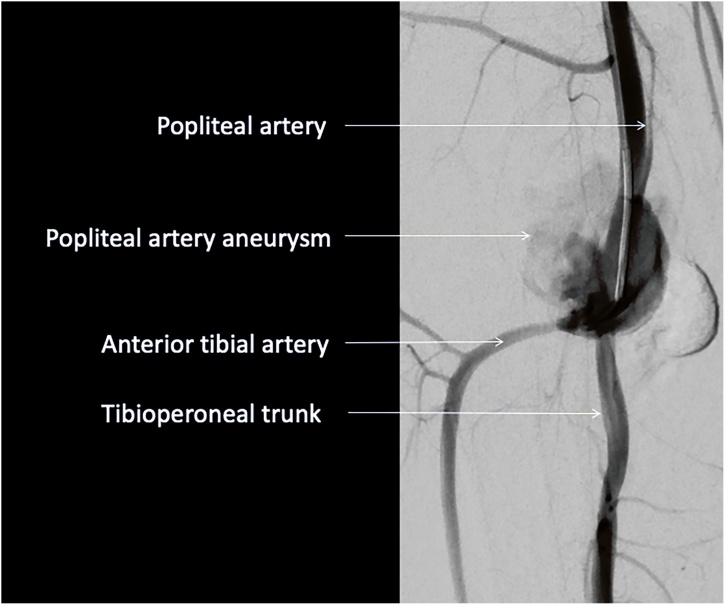
Angiogram of the knee with catheter close to popliteal artery defect showing aneurysm and runoff.

Clinical examination confirmed a tender pulsatile mass in the right popliteal fossa, with unilateral oedema to the right calf. Peripheral pulses were normal in both legs. There were no other palpable aneurysms present in any of the other major palpable arteries. Computed tomography angiography demonstrated a right popliteal pseudoaneurysm, measuring 3 × 4 × 4 cm (Fig. 1). Diagnosis of a pseudoaneurysm was based on a number of radiological findings. Firstly, the wall of the popliteal artery was visible behind the aneurysm; in contrast to a true aneurysm where there is a noticeable deviation of the vessel wall. Secondly, the angle between aneurysm was acute and sharp rather than obtuse and gradual, as seen in a true aneurysm. Following the diagnosis there was discussion surrounding the decision to proceed with surgical vs. endovascular management.

**Fig. 2:**
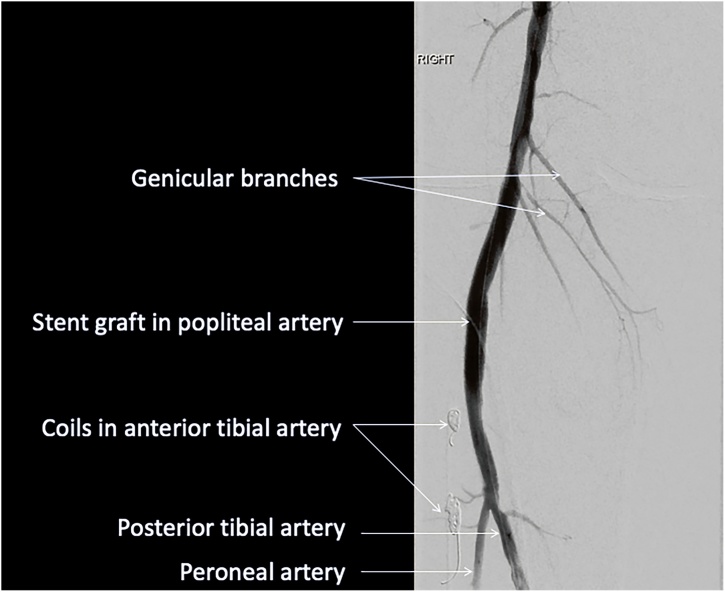
Post procedure angiogram shows successful exclusion of the aneurysm and sacrifice of the anterior tibial artery.

Given that the patient had a limited prognosis, and the requirement for palliative chemotherapy to prevent progression of malignancy, a decision was made to proceed with an endovascular approach and stent grafting of the popliteal artery. Diagnostic angiography showed a distal popliteal-tibioperoneal trunk false aneurysm with medium velocity anterior tibial artery flow and slow flow in the posterior tibial artery to the foot. The anterior tibial artery was embolised using 3 Cook® Retracta® detachable embolization coils. Following embolization, a 6 × 50 mm Gore® Viabahn® Endoprosthesis was inserted to the tibioperoneal trunk and a 7 × 50 mm Gore® Viabahn® Endoprosthesis was inserted to the distal popliteal artery, with a 10 mm overlap. Following stent insertion, angiography was repeated which demonstrated aneurysm exclusion and satisfactory runoff (Fig. 2). The patient was discharged after 48-h without complication. Informed and written patient consent was provided from the patient.

## 3 Discussion

This case is unusual in its presentation, given the lack of a definitive causative pathogenesis of the pseudoaneurysm. The combination of the large size of the pseudoaneurysm, rapid enlargement and onset of symptoms, along with a negative family and cardiovascular history negated a cardiovascular or atherosclerotic aetiology. The patient’s pathology results demonstrated no elevation of inflammatory markers or white cell count, which along with the clinical picture ruled out infective or mycotic pathogenesis as a cause for the rapid expansion of the pseudoaneurysm. The entity of popliteal artery entrapment syndrome was considered, however this syndrome usually presents in young sportsmen or soldiers with well-developed muscles, where exercise results in enlargement of muscles adjacent to the popliteal artery. In our case, the patient was older, there was no history of a sport or overuse mechanism of injury, the timeline was subacute in its onset, and the relationship of the time course of symptoms and chemotherapy excluded this as a differential diagnosis. More importantly, aneurysm formation as a complication of popliteal entrapment syndrome would be expected to occur higher up in the leg. This diagnosis was considered but the lesion was in the incorrect anatomical location for it to be a plausible differential diagnosis.

Taking into consideration the timeline of progression of the pseudoaneurysm, which presented 5 days after completion of four two-weekly cycles of FOLFOX6, and given that our patients pseudoaneurysm did not feature signs of inflammatory, mycotic or degenerative processes, with no history of penetrating trauma, we considered if the aneurysm may have developed due to treatment with FOLFIRI or FOLFOX chemotherapy. Previous anecdotal evidence suggesting a link between chemotherapy and aneurysm development exists. Two separate cases have reported acute enlargement of abdominal aortic aneurysms following cisplatin and 5-fluorouracil (5-FU) treatment [15,16]. An additional case report notes the formation of an intercostal artery pseudoaneurysm following trans-arterial administration of irinotecan, a key component of FOLFIRI chemotherapy [14]. Agents such as doxorubicin have been shown to compromise the integrity of the vascular wall through apoptosis of endothelial and smooth muscle cells [18]. Despite having differing mechanisms of action to doxorubicin, irinotecan, oxaliplatin, and 5-FU all lead to cell apoptosis [[19], [20], [21]], and thus may similarly play a role in compromising vascular integrity. High-dose irinotecan has been shown to increase necrosis and inflammation, resulting in reduced vessel wall thickness when used in drug-eluting stents in rabbit aortas [22]. Additionally, 5-FU has well established cardiotoxicity, including apical ballooning syndrome, which is thought to occur due to endothelial damage as well as coronary vascular compromise [20]. Given that in our case, the aneurysm developed acutely after treatment with FOLFOX, a mechanistic association is plausible. This raises the possibility that through apoptotic pathways, either or both FOLFIRI and FOLFOX chemotherapy compromise the integrity of the vessel wall, increasing the risk of aneurysm formation.

## 4 Conclusion

In conclusion, this case demonstrates the rapid occurrence of a spontaneous popliteal artery pseudoaneurysm in a man with metastatic colorectal cancer that developed acutely post FOLFOX chemotherapy. Diagnosis and prompt treatment of popliteal aneurysms are paramount to minimize inherent risks of complications, which include pain due to increased pressure from swelling or nerve compression, and extremity swelling due to venous compression. Major complications can also include limb-threatening ischaemia following thrombus formation and distal embolization. Differentiating aneurysms as false (pseudo) or true is important to help determine the underlying aetiology. Causes of pseudoaneurysms include arterial trauma e.g. arterial access for catheterization, and blunt or penetrating trauma. In contrast, true aneurysms commonly develop from inflammatory atherosclerosis, however other causes such as mycotic infection, inflammatory arteritis, and entrapment syndrome should be excluded. There may be some evidence to suggest a genetic predisposition to PAAs and anecdotal evidence exists suggests a relationship between chemotherapy and aneurysm progression, warranting further investigation into a possible causative link.

## Funding

This research did not receive any specific grant from funding agencies in the public, commercial, or not-for-profit sectors.

## Ethical approval

Austin Health Research Ethics committee has approved this report being submitted for publication. Written informed consent has been obtained by the patient and is available upon request from the corresponding author.

## Consent

Written informed consent was obtained from the patient for publication of this case report and accompanying images. A copy of the written consent is available for review by the Editor-in-Chief of this journal on request.

## Author’s contribution

A/Prof Laurence Weinberg: responsible for data collection, data interpretation, collation of all images and writing of the paper. He was the principal anaesthetist caring for the patient. He is the corresponding author and responsible for patient consent. Dr Manfred Spanger was the radiologist who interpreted the radiological scans. He was responsible for interpretation of all radiological images, data interpretation and writing of the paper. Dr Mayo Theivendren was the principal surgeon that managed the patient He was responsible for data interpretation, interpretation of all images and writing of the paper. Dr Luka Cosic was responsible for data collection, data interpretation, and writing of the paper. All authors were involved in drafting the article. All authors have read the final manuscript and approved it for submission.

## Registration of research studies

Not applicable.

## Guarantor

A/Prof Laurence Weinberg is the guarantor.

## Provenance and peer review

Not commissioned, externally peer-reviewed.

## Declaration of Competing Interest

The authors have no conflicts of interest to declare.
